# Propane-1,2-diaminium bis­(4-meth­oxy­benzoate)

**DOI:** 10.1107/S1600536811023968

**Published:** 2011-06-25

**Authors:** Zong-Ling Ru

**Affiliations:** a Department of Chemical & Environmental Engineering, Anyang Institute of Technology, Anyang 455000, People’s Republic of China

## Abstract

The asymmetric unit of the title salt, C_3_H_12_N_2_^2+^·2C_8_H_7_O_3_^−^, contains two 4-meth­oxy­benzoate anions and one propane-1,2-diaminium cation. All the amino H atoms of the cation are involved in N—H⋯O hydrogen bonds with the carboxyl­ate O atoms of the anions.

## Related literature

For related amide-acid co-crystal compounds, see: Almarsson & Zaworotko (2004 ▶); Blagden *et al.* (2008 ▶); Vishweshwar *et al.* (2006 ▶); Kapildev *et al.* (2011 ▶); Schultheiss & Newman (2009 ▶).
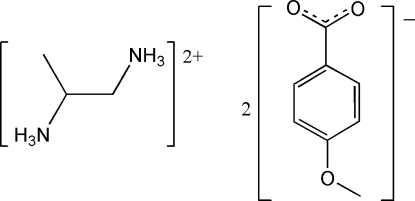


## Experimental

### 

#### Crystal data

C_3_H_12_N_2_^2+^·2C_8_H_7_O_3_^−^
*M* *_r_* = 378.42
Monoclinic, 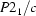

*a* = 13.847 (3) Å
*b* = 11.296 (2) Å
*c* = 12.893 (3) Å
β = 92.38 (3)°
*V* = 2014.8 (7) Å^3^
*Z* = 4
Mo *K*α radiation
μ = 0.09 mm^−1^
*T* = 298 K
0.30 × 0.05 × 0.05 mm

#### Data collection

Rigaku Mercury2 diffractometer
20478 measured reflections
4611 independent reflections
2264 reflections with *I* > 2σ(*I*)
*R*_int_ = 0.116

#### Refinement

*R*[*F* ^2^ > 2σ(*F* ^2^)] = 0.073
*wR*(*F* ^2^) = 0.194
*S* = 1.03
4611 reflections
244 parameters
H-atom parameters constrained
Δρ_max_ = 0.16 e Å^−3^
Δρ_min_ = −0.26 e Å^−3^

### 

Data collection: *CrystalClear* (Rigaku, 2005 ▶); cell refinement: *CrystalClear*; data reduction: *CrystalClear*; program(s) used to solve structure: *SHELXTL* (Sheldrick, 2008 ▶); program(s) used to refine structure: *SHELXTL*; molecular graphics: *SHELXTL*; software used to prepare material for publication: *SHELXTL*.

## Supplementary Material

Crystal structure: contains datablock(s) I, global. DOI: 10.1107/S1600536811023968/xu5245sup1.cif

Structure factors: contains datablock(s) I. DOI: 10.1107/S1600536811023968/xu5245Isup2.hkl

Supplementary material file. DOI: 10.1107/S1600536811023968/xu5245Isup3.cml

Additional supplementary materials:  crystallographic information; 3D view; checkCIF report

## Figures and Tables

**Table 1:** Hydrogen-bond geometry (Å, °)

*D*—H⋯*A*	*D*—H	H⋯*A*	*D*⋯*A*	*D*—H⋯*A*
N1—H1*A*⋯O4^i^	0.90	1.82	2.696 (3)	162
N1—H1*B*⋯O1	0.90	1.92	2.791 (3)	162
N1—H1*C*⋯O1^ii^	0.90	1.89	2.777 (3)	167
N2—H2*A*⋯O5^iii^	0.90	1.82	2.718 (3)	173
N2—H2*B*⋯O4^iv^	0.90	2.03	2.917 (3)	170
N2—H2*C*⋯O2^ii^	0.90	1.81	2.703 (3)	1670

Symmetry codes: (i) 
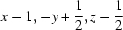
; (ii) 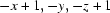; (iii) 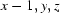; (iv) 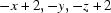.

**Fig. 1.**
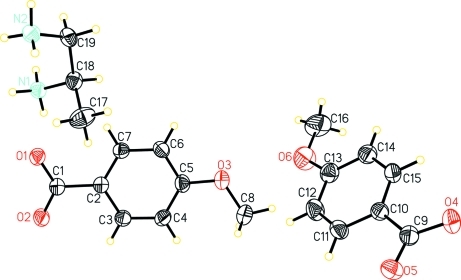
Molecular view of the title compound with the atomic numbering scheme. Displacement ellipsoids are drawn at the 30% probability level.

**Fig. 2.**
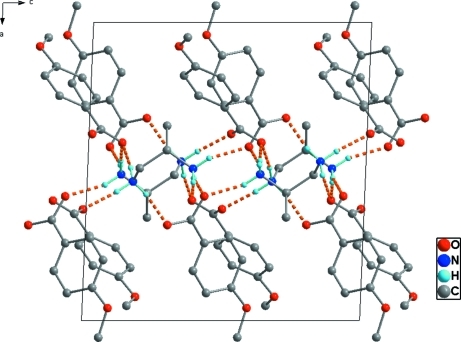
The crystal packing of the title compound viewed along the b axis showing the two-dimensional hydrogen bondings network (dashed line). Hydrogen atoms not involved in hydrogen bonding have been omitted for clarity.

## supplementary crystallographic information

### Comment

Molecular cocrystals are becoming increasingly important within the pharmaceutical industry as an alternative source of new solid crystalline materials with the potential to provide optimal physical properties whilst retaining the chemical properties of the cocrystal components (Almarsson & Zaworotko, 2004; Blagden *et al.*, 2008; Vishweshwar *et al.*, 2006). Physicochemical properties such as the melting point, stability and solubility of an active pharmaceutical ingredient can be tuned through cocrystal formulation (Kapildev *et al.*, 2011; Schultheiss & Newman, 2009). Cocrystal synthesis often relies on the acid-amide H-bonds interactions. Herein, we report the crystal structure of the title compound, propane-1,2-diaminium di-4-methoxybenzoate.

The asymmetric unit is composed of two 4-methoxybenzoate anions and one propane-1,2-diaminium cation (Fig. 1). Both the amine N atoms were protonated. And the carboxyl groups were deprotonated. The geometric parameters of the title compound are in the normal range.

In the crystal structure, all the amino H atoms are involved in N—H···O hydrogen bonds with the carboxyl O atoms (Table 1 and Fig. 2).

### Experimental

A mixture of *R*-propane-1,2-diamine (1.0 mmol), 4-methoxybenzoic acid (2.0 mmol) and 20 ml ethanol were added into a 50 ml flask and refluxed for 5 h, then cooled and filtrated. The solution was evaporated slowly in the air. Colorless block crystals suitable for X-ray analysis were obtained after one week. The *R*-propane-1,2-diamine turned into racemic amine in the heating process.

### Refinement

All H atoms attached to C atoms were fixed geometrically and treated as riding with C—H = 0.98 (methine), 0.97 (methylene), 0.96 (methyl) and 0.93 Å (aromatic), *U*_iso_(H) = 1.2*U*_eq_(C except methyl) and *U*_iso_(H) = 1.5*U*_eq_(C of methyl). The amino H atoms were placed in calculated positions and refined in riding mode with U_iso_(H) = 1.5U_eq_(N).

### Crystal data

**Table:**

C_3_H_12_N_2_^2+^·2C_8_H_7_O_3_^−^	*F*(000) = 808
*M**_r_* = 378.42	*D*_x_ = 1.247 Mg m^−^^3^
Monoclinic, *P*2_1_/*c*	Mo *K*α radiation, λ = 0.71073 Å
Hall symbol: -P 2ybc	Cell parameters from 4611 reflections
*a* = 13.847 (3) Å	θ = 3.2–27.5°
*b* = 11.296 (2) Å	µ = 0.09 mm^−^^1^
*c* = 12.893 (3) Å	*T* = 298 K
β = 92.38 (3)°	Block, colorless
*V* = 2014.8 (7) Å^3^	0.30 × 0.05 × 0.05 mm
*Z* = 4	

### Data collection

**Table:**

Rigaku Mercury2 diffractometer	2264 reflections with *I* > 2σ(*I*)
Radiation source: fine-focus sealed tube	*R*_int_ = 0.116
graphite	θ_max_ = 27.5°, θ_min_ = 3.2°
Detector resolution: 13.6612 pixels mm^-1^	*h* = −17→17
CCD profile fitting scans	*k* = −14→14
20478 measured reflections	*l* = −16→16
4611 independent reflections	

### Refinement

**Table:**

Refinement on *F*^2^	Primary atom site location: structure-invariant direct methods
Least-squares matrix: full	Secondary atom site location: difference Fourier map
*R*[*F*^2^ > 2σ(*F*^2^)] = 0.073	Hydrogen site location: inferred from neighbouring sites
*wR*(*F*^2^) = 0.194	H-atom parameters constrained
*S* = 1.03	*w* = 1/[σ^2^(*F*_o_^2^) + (0.0691*P*)^2^ + 0.210*P*] where *P* = (*F*_o_^2^ + 2*F*_c_^2^)/3
4611 reflections	(Δ/σ)_max_ < 0.001
244 parameters	Δρ_max_ = 0.16 e Å^−^^3^
0 restraints	Δρ_min_ = −0.26 e Å^−^^3^

### Special details

**Table:**

Geometry. All esds (except the esd in the dihedral angle between two l.s. planes) are estimated using the full covariance matrix. The cell esds are taken into account individually in the estimation of esds in distances, angles and torsion angles; correlations between esds in cell parameters are only used when they are defined by crystal symmetry. An approximate (isotropic) treatment of cell esds is used for estimating esds involving l.s. planes.
Refinement. Refinement of F^2^ against ALL reflections. The weighted R-factor wR and goodness of fit S are based on F^2^, conventional R-factors R are based on F, with F set to zero for negative F^2^. The threshold expression of F^2^ > 2sigma(F^2^) is used only for calculating R-factors(gt) etc. and is not relevant to the choice of reflections for refinement. R-factors based on F^2^ are statistically about twice as large as those based on F, and R- factors based on ALL data will be even larger.

### Fractional atomic coordinates and isotropic or equivalent isotropic displacement parameters (Å^2^)

**Table:**

	*x*	*y*	*z*	*U*_iso_*/*U*_eq_	
O1	0.58273 (13)	0.11367 (16)	0.42325 (14)	0.0557 (5)	
O4	1.40303 (14)	0.20038 (18)	1.12055 (15)	0.0653 (6)	
O5	1.37392 (14)	0.05243 (18)	1.01087 (14)	0.0620 (6)	
N1	0.50945 (15)	0.09975 (19)	0.62133 (15)	0.0498 (6)	
H1A	0.4635	0.1562	0.6181	0.075*	
H1B	0.5442	0.1111	0.5647	0.075*	
H1C	0.4780	0.0300	0.6173	0.075*	
C10	1.28462 (19)	0.2302 (2)	0.9837 (2)	0.0501 (7)	
O2	0.67887 (14)	0.0757 (2)	0.29455 (15)	0.0690 (6)	
N2	0.46696 (16)	−0.00178 (19)	0.83553 (16)	0.0512 (6)	
H2A	0.4327	0.0114	0.8922	0.077*	
H2B	0.5128	−0.0577	0.8457	0.077*	
H2C	0.4235	−0.0264	0.7863	0.077*	
C2	0.73928 (19)	0.2000 (2)	0.4300 (2)	0.0467 (7)	
C1	0.6619 (2)	0.1256 (2)	0.3787 (2)	0.0477 (7)	
C9	1.3588 (2)	0.1555 (3)	1.0418 (2)	0.0535 (7)	
O3	0.95714 (15)	0.3848 (2)	0.59171 (17)	0.0752 (7)	
C7	0.7231 (2)	0.2669 (2)	0.5179 (2)	0.0520 (7)	
H7A	0.6613	0.2694	0.5432	0.062*	
C18	0.57151 (19)	0.1152 (3)	0.7178 (2)	0.0536 (8)	
H18A	0.6003	0.1942	0.7140	0.064*	
O6	1.08060 (18)	0.4243 (2)	0.81081 (19)	0.0930 (8)	
C6	0.7958 (2)	0.3295 (3)	0.5685 (2)	0.0584 (8)	
H6A	0.7825	0.3749	0.6264	0.070*	
C14	1.1855 (2)	0.4049 (3)	0.9672 (3)	0.0634 (8)	
H14A	1.1651	0.4766	0.9943	0.076*	
C3	0.8322 (2)	0.2004 (3)	0.3933 (2)	0.0578 (8)	
H3A	0.8447	0.1583	0.3333	0.069*	
C19	0.5112 (2)	0.1142 (2)	0.8128 (2)	0.0538 (7)	
H19A	0.5516	0.1382	0.8723	0.065*	
H19B	0.4601	0.1725	0.8034	0.065*	
C4	0.9066 (2)	0.2618 (3)	0.4437 (2)	0.0621 (8)	
H4A	0.9683	0.2608	0.4177	0.074*	
C15	1.2534 (2)	0.3372 (3)	1.0210 (2)	0.0556 (8)	
H15A	1.2790	0.3645	1.0844	0.067*	
C11	1.2458 (2)	0.1912 (3)	0.8880 (2)	0.0610 (8)	
H11A	1.2658	0.1192	0.8612	0.073*	
C5	0.8888 (2)	0.3252 (3)	0.5335 (2)	0.0578 (8)	
C13	1.1480 (2)	0.3652 (3)	0.8723 (3)	0.0643 (8)	
C12	1.1784 (2)	0.2581 (3)	0.8329 (2)	0.0695 (9)	
H12A	1.1532	0.2313	0.7691	0.083*	
C8	1.0557 (2)	0.3755 (3)	0.5628 (3)	0.0838 (11)	
H8A	1.0963	0.4209	0.6101	0.126*	
H8B	1.0616	0.4054	0.4936	0.126*	
H8C	1.0754	0.2940	0.5653	0.126*	
C17	0.6536 (2)	0.0284 (3)	0.7205 (3)	0.0834 (11)	
H17A	0.6934	0.0407	0.7823	0.125*	
H17B	0.6915	0.0397	0.6606	0.125*	
H17C	0.6284	−0.0508	0.7203	0.125*	
C16	1.0508 (3)	0.5392 (4)	0.8406 (3)	0.1064 (14)	
H16A	1.0038	0.5689	0.7902	0.160*	
H16B	1.1057	0.5911	0.8443	0.160*	
H16C	1.0227	0.5352	0.9073	0.160*	

### Atomic displacement parameters (Å^2^)

**Table:**

	*U*^11^	*U*^22^	*U*^33^	*U*^12^	*U*^13^	*U*^23^
O1	0.0547 (12)	0.0615 (13)	0.0513 (11)	−0.0116 (10)	0.0062 (10)	−0.0012 (9)
O4	0.0720 (14)	0.0648 (13)	0.0573 (12)	−0.0055 (11)	−0.0173 (10)	−0.0007 (11)
O5	0.0738 (14)	0.0605 (13)	0.0516 (12)	0.0096 (11)	0.0034 (10)	−0.0024 (11)
N1	0.0553 (14)	0.0550 (14)	0.0392 (12)	−0.0071 (11)	0.0029 (11)	−0.0012 (11)
C10	0.0513 (17)	0.0542 (18)	0.0448 (15)	−0.0003 (14)	0.0028 (13)	−0.0006 (14)
O2	0.0651 (14)	0.0942 (16)	0.0480 (12)	−0.0191 (11)	0.0073 (10)	−0.0194 (11)
N2	0.0559 (14)	0.0587 (15)	0.0387 (12)	−0.0003 (12)	−0.0003 (11)	−0.0010 (11)
C2	0.0501 (17)	0.0468 (16)	0.0430 (15)	−0.0013 (13)	−0.0011 (13)	0.0056 (13)
C1	0.0533 (18)	0.0490 (17)	0.0405 (16)	−0.0041 (14)	−0.0017 (14)	0.0079 (13)
C9	0.0532 (18)	0.066 (2)	0.0416 (16)	−0.0069 (16)	0.0064 (14)	0.0029 (15)
O3	0.0579 (14)	0.0904 (17)	0.0763 (15)	−0.0086 (12)	−0.0097 (11)	−0.0223 (13)
C7	0.0525 (17)	0.0523 (17)	0.0513 (16)	0.0005 (14)	0.0048 (14)	0.0006 (14)
C18	0.0538 (18)	0.0624 (19)	0.0442 (16)	−0.0064 (15)	−0.0028 (14)	0.0030 (14)
O6	0.0970 (18)	0.0944 (18)	0.0850 (17)	0.0279 (15)	−0.0254 (14)	0.0055 (14)
C6	0.061 (2)	0.0561 (18)	0.0574 (18)	0.0016 (15)	−0.0010 (16)	−0.0108 (15)
C14	0.059 (2)	0.0568 (19)	0.074 (2)	0.0010 (16)	0.0023 (17)	−0.0053 (17)
C3	0.0562 (19)	0.070 (2)	0.0472 (16)	−0.0062 (16)	0.0072 (14)	−0.0060 (15)
C19	0.0635 (18)	0.0525 (18)	0.0447 (16)	−0.0050 (14)	−0.0041 (14)	−0.0028 (14)
C4	0.0476 (17)	0.081 (2)	0.0580 (18)	−0.0044 (16)	0.0071 (15)	−0.0069 (17)
C15	0.0541 (18)	0.0596 (19)	0.0526 (17)	−0.0054 (15)	−0.0050 (14)	−0.0059 (15)
C11	0.0656 (19)	0.067 (2)	0.0497 (17)	0.0071 (16)	−0.0064 (15)	−0.0097 (16)
C5	0.0551 (19)	0.0623 (19)	0.0549 (18)	−0.0030 (15)	−0.0094 (15)	−0.0046 (15)
C13	0.060 (2)	0.064 (2)	0.068 (2)	0.0044 (16)	−0.0051 (17)	0.0044 (18)
C12	0.077 (2)	0.077 (2)	0.0534 (18)	0.0050 (18)	−0.0186 (17)	−0.0039 (17)
C8	0.056 (2)	0.102 (3)	0.092 (3)	−0.0092 (19)	−0.0091 (18)	−0.018 (2)
C17	0.066 (2)	0.109 (3)	0.076 (2)	0.021 (2)	0.0092 (18)	0.022 (2)
C16	0.105 (3)	0.093 (3)	0.121 (3)	0.047 (2)	−0.004 (3)	0.013 (3)

### Geometric parameters (Å, °)

**Table:**

O1—C1	1.266 (3)	O6—C16	1.420 (4)
O4—C9	1.269 (3)	C6—C5	1.383 (4)
O5—C9	1.250 (3)	C6—H6A	0.9300
N1—C18	1.492 (3)	C14—C15	1.377 (4)
N1—H1A	0.9004	C14—C13	1.384 (4)
N1—H1B	0.9002	C14—H14A	0.9300
N1—H1C	0.9004	C3—C4	1.381 (4)
C10—C15	1.377 (4)	C3—H3A	0.9300
C10—C11	1.396 (4)	C19—H19A	0.9700
C10—C9	1.505 (4)	C19—H19B	0.9700
O2—C1	1.253 (3)	C4—C5	1.393 (4)
N2—C19	1.481 (3)	C4—H4A	0.9300
N2—H2A	0.9003	C15—H15A	0.9300
N2—H2B	0.9006	C11—C12	1.375 (4)
N2—H2C	0.9005	C11—H11A	0.9300
C2—C7	1.387 (4)	C13—C12	1.385 (4)
C2—C3	1.390 (4)	C12—H12A	0.9300
C2—C1	1.495 (4)	C8—H8A	0.9600
O3—C5	1.362 (3)	C8—H8B	0.9600
O3—C8	1.434 (3)	C8—H8C	0.9600
C7—C6	1.372 (4)	C17—H17A	0.9600
C7—H7A	0.9300	C17—H17B	0.9600
C18—C17	1.500 (4)	C17—H17C	0.9600
C18—C19	1.511 (4)	C16—H16A	0.9600
C18—H18A	0.9800	C16—H16B	0.9600
O6—C13	1.372 (4)	C16—H16C	0.9600
			
C18—N1—H1A	109.7	C2—C3—H3A	119.2
C18—N1—H1B	110.5	N2—C19—C18	114.4 (2)
H1A—N1—H1B	105.2	N2—C19—H19A	108.6
C18—N1—H1C	114.2	C18—C19—H19A	108.6
H1A—N1—H1C	106.1	N2—C19—H19B	108.6
H1B—N1—H1C	110.6	C18—C19—H19B	108.6
C15—C10—C11	118.1 (3)	H19A—C19—H19B	107.6
C15—C10—C9	122.4 (3)	C3—C4—C5	119.6 (3)
C11—C10—C9	119.5 (3)	C3—C4—H4A	120.2
C19—N2—H2A	104.5	C5—C4—H4A	120.2
C19—N2—H2B	110.8	C10—C15—C14	121.9 (3)
H2A—N2—H2B	113.0	C10—C15—H15A	119.0
C19—N2—H2C	113.8	C14—C15—H15A	119.0
H2A—N2—H2C	105.3	C12—C11—C10	120.8 (3)
H2B—N2—H2C	109.3	C12—C11—H11A	119.6
C7—C2—C3	117.4 (3)	C10—C11—H11A	119.6
C7—C2—C1	122.1 (2)	O3—C5—C6	116.0 (3)
C3—C2—C1	120.5 (3)	O3—C5—C4	124.7 (3)
O2—C1—O1	122.8 (3)	C6—C5—C4	119.3 (3)
O2—C1—C2	118.7 (2)	O6—C13—C14	125.1 (3)
O1—C1—C2	118.5 (2)	O6—C13—C12	115.0 (3)
O5—C9—O4	123.1 (3)	C14—C13—C12	119.9 (3)
O5—C9—C10	118.9 (3)	C11—C12—C13	120.0 (3)
O4—C9—C10	118.1 (3)	C11—C12—H12A	120.0
C5—O3—C8	117.9 (2)	C13—C12—H12A	120.0
C6—C7—C2	122.0 (3)	O3—C8—H8A	109.5
C6—C7—H7A	119.0	O3—C8—H8B	109.5
C2—C7—H7A	119.0	H8A—C8—H8B	109.5
N1—C18—C17	110.7 (2)	O3—C8—H8C	109.5
N1—C18—C19	110.9 (2)	H8A—C8—H8C	109.5
C17—C18—C19	114.9 (2)	H8B—C8—H8C	109.5
N1—C18—H18A	106.7	C18—C17—H17A	109.5
C17—C18—H18A	106.7	C18—C17—H17B	109.5
C19—C18—H18A	106.7	H17A—C17—H17B	109.5
C13—O6—C16	119.1 (3)	C18—C17—H17C	109.5
C7—C6—C5	120.0 (3)	H17A—C17—H17C	109.5
C7—C6—H6A	120.0	H17B—C17—H17C	109.5
C5—C6—H6A	120.0	O6—C16—H16A	109.5
C15—C14—C13	119.3 (3)	O6—C16—H16B	109.5
C15—C14—H14A	120.4	H16A—C16—H16B	109.5
C13—C14—H14A	120.4	O6—C16—H16C	109.5
C4—C3—C2	121.6 (3)	H16A—C16—H16C	109.5
C4—C3—H3A	119.2	H16B—C16—H16C	109.5

### Hydrogen-bond geometry (Å, °)

**Table:**

*D*—H···*A*	*D*—H	H···*A*	*D*···*A*	*D*—H···*A*
N1—H1A···O4^i^	0.90	1.82	2.696 (3)	162
N1—H1B···O1	0.90	1.92	2.791 (3)	162
N1—H1C···O1^ii^	0.90	1.89	2.777 (3)	167
N2—H2A···O5^iii^	0.90	1.82	2.718 (3)	173
N2—H2B···O4^iv^	0.90	2.03	2.917 (3)	170
N2—H2C···O2^ii^	0.90	1.81	2.703 (3)	1670

Symmetry codes: (i) *x*−1, −*y*+1/2, *z*−1/2; (ii) −*x*+1, −*y*, −*z*+1; (iii) *x*−1, *y*, *z*; (iv) −*x*+2, −*y*, −*z*+2.
